# Diabetic Mastopathy With an Associated History of Elevated Thyroid Peroxidase (TPO) and Antimitochondrial Antibodies (AMA)

**DOI:** 10.7759/cureus.37299

**Published:** 2023-04-08

**Authors:** Shreyan A Patel, Saba Naamo, Liping Li, Tatianie Jackson

**Affiliations:** 1 Internal Medicine, Edward Via College of Osteopathic Medicine, Blacksburg, USA; 2 Radiology, Geisinger Medical Center, Danville, USA; 3 Pathology, Geisinger Medical Center, Danville, USA

**Keywords:** autoimmune, breast mass, diabetes mellitus, type 1 diabetes mellitus, fibrous mastopathy, diabetic mastopathy

## Abstract

Diabetic mastopathy is an uncommon, benign breast lesion that is typically seen in patients with type 1 diabetes mellitus (T1DM). The main differential diagnosis for diabetic mastopathy is breast carcinoma, which appears similarly on clinical examination and diagnostic imaging. Although the etiology of diabetic mastopathy is poorly understood, it has been associated with several autoimmune conditions, such as Hashimoto’s thyroiditis, systemic lupus erythematosus, and Sjogren’s syndrome. Here, we report a case of diabetic mastopathy in a T1DM patient with an associated history of elevated thyroid peroxidase (TPO) and antimitochondrial antibody (AMA) levels, giving further support to the theory of autoimmune etiopathogenesis.

## Introduction

Diabetic mastopathy, also known as fibrous mastopathy, is a rare condition that comprises less than 1% of benign breast pathologies and is a known complication of type 1 diabetes mellitus (T1DM) [1]. This disease affects 0.6% to 13% of young and middle-aged premenopausal females with T1DM, but there have been infrequent reports of diabetic mastopathy occurring in patients with type 2 diabetes mellitus, the elderly, and males [1-4]. Diabetic mastopathy was first described by Sloer and Khardori in 1984 and was officially named by Logan and Hoffman in 1989 [5,6]. Although the mechanism by which diabetic mastopathy arises is not fully understood, it has been hypothesized that there may be autoimmune factors contributing to its pathogenesis [4].

## Case presentation

A 37-year-old female with a past medical history of T1DM, Hashimoto’s thyroiditis, stage 3 chronic kidney disease, dyslipidemia, chronic obstructive pulmonary disease, and smoking presented with an enlarging left breast mass in the 12 o’clock position. She first noticed the mass eight months ago, but it recently became painful after sustaining mild trauma to the area. She denied nipple discharge or changes in the characteristics of the mass with menstruation.

Her last mammogram two years ago was negative (BI-RADS 1). She had a history of pseudoangiomatous stromal hyperplasia in the right breast, for which she underwent a lumpectomy. She was premenopausal and nulliparous; menarche occurred at the age of 17. She previously took oral contraceptive pills and had been receiving intramuscular medroxyprogesterone injections for abnormal uterine bleeding for the past eight years. Her family history was significant for breast cancer in her mother, paternal aunt, paternal grandmother, and paternal great-grandmother.

**Figure 1:**
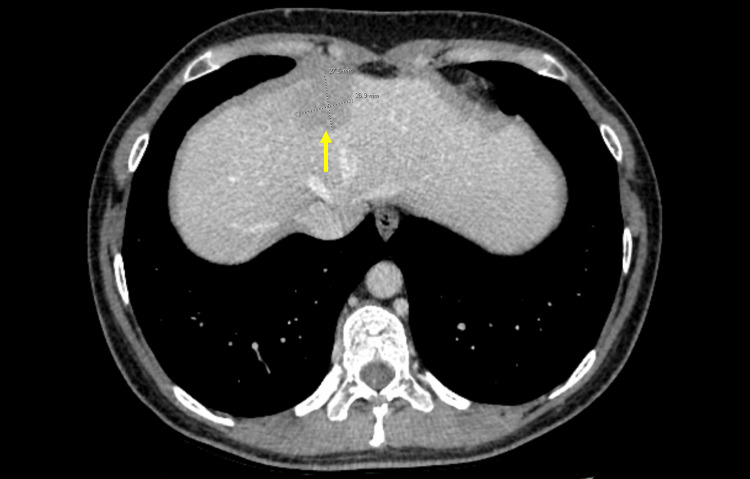
CT abdomen scan showing an incidental 2.8 cm, low-attenuating, hypodense lesion in the right hepatic lobe between segments VIII and IVa (yellow arrow), likely representing a vascular shunt or adenoma.

She was diagnosed with T1DM at the age of five and had been on an insulin pump for the past 16 years. Her most recent hemoglobin A1c was 9.6% but had been as high as 14% in the past. She had significant complications from T1DM, which include retinopathy, polyneuropathy, nephropathy, neurogenic bladder, and gastroparesis, resulting in pyloroplasty and gastric pacemaker placement. Her TPO antibody level from five years ago was elevated at 230.2 IU/mL (normal < 34 IU/mL), but she continued to remain euthyroid; her most recent TSH level from eight months ago was within normal limits. She also had a history of elevated transaminase levels to two times the upper normal limit with positive AMA antibodies three years ago; however, a liver biopsy at the time showed mild, nonspecific inflammation. She underwent a computed tomography (CT) abdomen scan one month ago, which incidentally revealed a 2.8 cm low-attenuating, hypodense lesion in the right hepatic lobe between segments VIII and IVa, which likely represented a vascular shunt or adenoma (Figure 1).

**Figure 2:**
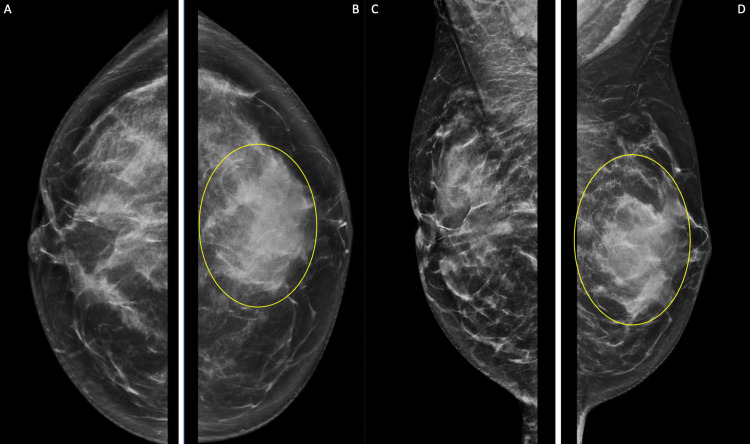
Diagnostic mammogram with (a) craniocaudal view of the right breast and (b) craniocaudal view of the left breast (c) mediolateral oblique view of the right breast (d) mediolateral oblique view of the left breast. The left breast is heterogeneously dense with a focal asymmetry in the retroareolar region, deep to the palpable abnormality (yellow ovals).

**Figure 3:**
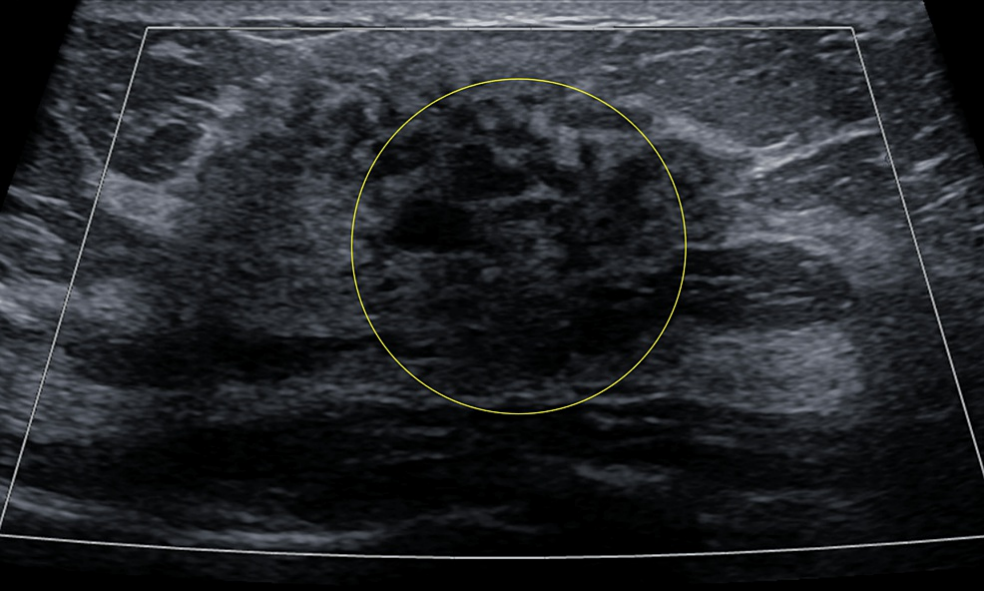
Transverse view ultrasound of the left breast showed a hypoechoic area at the 12 o’clock position with no internal vascularity (yellow circle).

**Figure 4:**
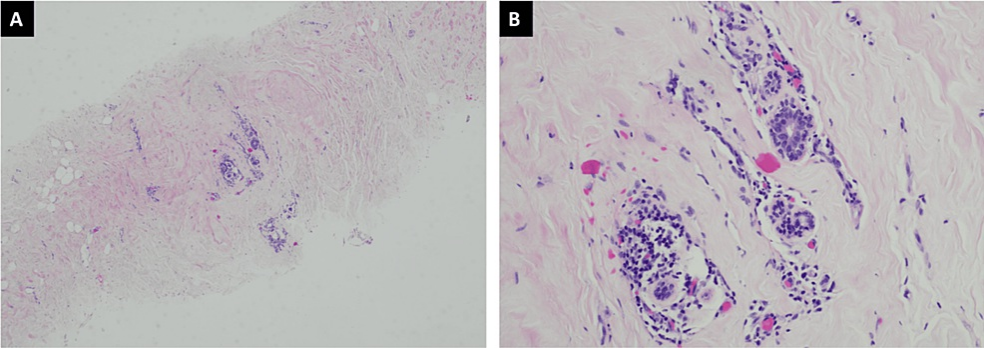
Lymphocytic mastopathy (A) H&E demonstrates hyalinized interlobular stroma at 40x and (B) dense lymphocytic infiltrates that are centered around ducts, lobules, and blood vessels at 200x.

**Figure 5:**
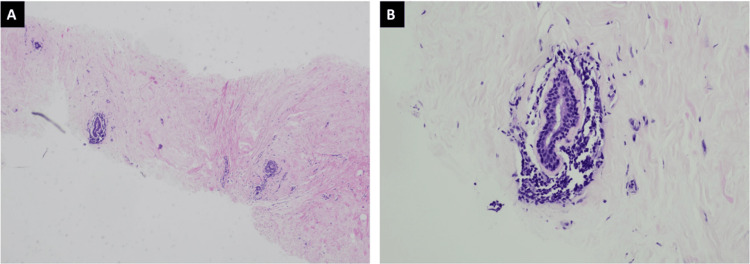
Lymphocytic mastopathy (A) H&E demonstrates hyalinized interlobular stroma at 40x and (B) dense lymphocytic infiltrates that are centered around ducts and blood vessels at 200x.

Diagnostic mammography showed a heterogeneously dense left breast with a focal asymmetry in the retroareolar region deep to the palpable abnormality; the right breast was also heterogeneously dense without any mammographic evidence of malignancy (Figure 2). Focused left breast ultrasound demonstrated heterogeneous background echotexture with a hypoechoic area spanning 67 mm at the 12 o’clock position (Figure 3). The lesion was characterized as BI-RADS 4. Ultrasound-guided core biopsy results revealed fibrotic stroma with keloidal features and tiny clusters of lymphocytic infiltrate around small vessels without any evidence of carcinoma (Figures 4-5).

## Discussion

Diagnostic mammography is usually nonspecific in the presence of dense glandular breast tissue [4]. In the case of diabetic mastopathy, focused ultrasound examination routinely shows a hypoechoic area with posterior acoustic shadowing [2]. However, since patients present with a painless, palpable, and irregular breast mass, the clinical examination and diagnostic imaging findings are concerning for breast carcinoma, which must be excluded using histopathological analysis. Although our patient’s focused left breast ultrasound demonstrated an abnormal hypoechoic area, she had a strong family history of breast cancer, which made her symptoms and findings even more suspicious for malignancy.

Pathological features for diabetic mastopathy include perivascular lymphocytic infiltrate of mature B cells, lobular atrophy, keloidal fibrosis, and myofibroblastic epithelioid cells [2]. Our patient’s ultrasound-guided core biopsy results were consistent with diabetic mastopathy. This was a typical case of diabetic mastopathy, which occurred in a premenopausal female with long-standing, poorly controlled, insulin-dependent diabetes mellitus, as seen by the presence of many microvascular complications and elevated hemoglobin A1c levels.

Although the exact pathogenesis of diabetic mastopathy is unknown, some hypotheses include anti-insulin antibodies cross-reacting with breast epithelium, leading to inflammation and fibrosis, and hyperglycemia causing increased collagen production, decreased collagen degradation, and subsequent extracellular matrix expansion [2,4]. Of note, the histopathological changes seen in diabetic mastopathy are also seen in other autoimmune conditions, such as Hashimoto’s thyroiditis, systemic lupus erythematosus, Sjogren’s syndrome, Addison’s disease, IgG-4-related disease [4]. Our patient had a history of elevated antibody levels consistent with Hashimoto’s thyroiditis and concern for primary biliary cholangitis (PBC). Although positive AMA antibodies are highly specific and sensitive for PBC, and this condition typically arises in middle-aged females, our patient did not have a consistent elevation of cholestatic enzymes, elevated alkaline phosphatase levels, or typical liver histology; this, in combination with the absence of fatigue, pruritus, and jaundice, makes the diagnosis of atypical PBC unlikely [7]. The presence of these coinciding autoimmune processes could corroborate theories of a possible autoimmune etiology for diabetic mastopathy, but further investigation is required.

The differential diagnosis for a hepatic lesion includes focal nodular hyperplasia, adenoma, hemangioma, hepatocellular carcinoma, and metastasis [8]. The incidental hypodense liver lesion seen in our patient’s CT abdomen scan was only visible during the portal venous phase of the four-phase liver CT protocol and, as a result, likely represented a vascular shunt or adenoma. Given our patient’s history of an enlarging breast mass and strong family history of breast cancer, it was important to rule out a metastatic lesion.

Treatment for diabetic mastopathy is mainly conservative [4]. There is no increased risk for malignant transformation of the lesion, nor is there an increased risk for developing breast carcinoma compared to the general population [2]. As a result, there is no role in surgical management unless malignancy cannot be definitively ruled out [4].

## Conclusions

Diabetic mastopathy is a rare breast condition most commonly seen in patients with type 1 diabetes mellitus. Since the clinical presentation and diagnostic imaging of diabetic mastopathy closely mimic those of breast carcinoma, it is important to have a high index of suspicion and to distinguish between these two diagnoses with histopathological analysis. Further research is required to better elucidate the etiopathogenesis of diabetic mastopathy, especially given its frequent association with other concurrent autoimmune disease processes.
